# Cytoplasmic transcription factor sequestration drives the pathogenesis of a rearranged leukemia

**DOI:** 10.1172/JCI179105

**Published:** 2024-02-15

**Authors:** Ali Shilatifard

The oncofusion genes created by chromosomal translocations frequently drive hematological malignancies. In general, the chimeric proteins resulting from these oncofusions initiate and drive cancer pathogenesis by disrupting specific cellular mechanisms regulating patterns of gene expression. For example, translocations involving chromosome 11q23, which are among the most common found in pediatric leukemias, create fusions of the MLL-encoding gene *KMT2A* with any of a large number of genes on other chromosomes. It was mechanistically demonstrated that several of these seemingly unrelated oncofusion partners encode subunits of an RNA polymerase II elongation factor now known as the super elongation complex (SEC) (1). Therefore, translocations involving chromosome 11q23 drive cancer by creating chimeric fusions of MLL to SEC subunits, disrupting SEC’s role in transcriptional elongation checkpoint control. In other cases, oncofusion protein chimeras may also affect transcription factor stability, alter patterns of chromatin binding, hijack enhancers, or otherwise disrupt transcriptional regulatory mechanisms.

In an elegant study from Tim Ley’s laboratory published recently in the *JCI*, Day et al. (2) defined another mechanism by which translocations can cause cancer, demonstrating that the CBFB::MYH1 oncofusion protein chimera drives oncogenesis by sequestering the transcription factor Runx1 in the cytoplasm.

The Runx family of transcription factors (Runx1, Runx2, and Runx3), which heterodimerize with mammalian core binding factor β (CBFB) to form the hematopoiesis regulator core binding factor (CBF), plays central roles in acute myeloid leukemias (AMLs) initiated by the *RUNX1::RUNX1T1* and *CBFB::MYH11* oncofusions (3). For *RUNX1::RUNX1T1* AML, recruitment of repressor machinery to genomic loci bound by CBF downregulates CBF target genes, driving oncogenesis (3). For *CBFB::MYH11* AML, however, the mechanisms driving pathogenesis were previously unclear. To address this, Day et al. (2) fused the TurboID promiscuous biotin ligase (4) to oncogenes in a proximity-based labeling strategy to identify protein factors interacting with protein complexes associated with the CBFB::MYH11 oncofusion protein.

This approach revealed numerous cytoplasmic proteins in the CBFB::MYH11-TurboID interactome, indicating that cytoplasmic CBFB::MYH11 retention that may occur due to interaction of MYH11 with myosin and related cytoplasmic proteins. Using molecular, biochemical, and cell biology methods, Day et al. (2) demonstrated that cytoplasmic CBFB::MYH11 retention in the cytoplasm resulted in sequestration of RUNX1 in the cytoplasm in a form of aggregates. RUNX1 aggregation due to CBFB::MYH11 retention in the cytosol was confirmed in primary human AML cells, suggesting that this is the actual mechanism of the disease in humans. Unexpectedly, Day et al. also found that CBFB::MYH11 increased expression of both RUNX1 and RUNX2 in the nucleus, suggesting the existence of an intriguing mechanism to sense and compensate for reduced CBF function.

Similar relocalization of transcription regulatory factors from the nucleus to the cytosol has previously been reported for solid tumors (5). It was recently reported that truncated mutant forms of MLL4, which is found in a large number of cancers and developmental disorders such as Kabuki syndrome, relocalize to the cytoplasm in animal models of cancer and samples from patients with bladder cancer (6). The elegant report of the cytoplasmic retention of the CBFB::MYH11 chimera and concomitant sequestration of the RUNX transcription factor in the *JCI* provides a mechanism by which oncofusion genes drive the pathogenesis of hematological malignancies, informing both prognosis and therapeutic development (2).

## Version 1 02/15/2024

Electronic publication

## References

[1] Lin C (2010). AFF4, a component of the ELL/P-TEFb elongation complex and a shared subunit of MLL chimeras, can link transcription elongation to leukemia. Mol Cell.

[2] Day RB (2023). Proteogenomic analysis reveals cytoplasmic sequestration of RUNX1 by the acute myeloid leukemia–initiating CBFB::MYH11 oncofusion protein. J Clin Invest.

[3] Beghini A (2019). Core binding factor leukemia: chromatin remodeling moves towards oncogenic transcription. Cancers (Basel).

[4] Branon TC (2018). Efficient proximity labeling in living cells and organisms with TurboID. Nat Biotechnol.

[5] Wang L (2017). A cytoplasmic COMPASS is necessary for cell survival and triple-negative breast cancer pathogenesis by regulating metabolism. Genes Dev.

[6] Zhao Z (2023). Somatic mutations of MLL4/COMPASS induce cytoplasmic localization providing molecular insight into cancer prognosis and treatment. Proc Natl Acad Sci U S A.

